# Hypogalactosylation of serum N-glycans fails to predict clinical response to methotrexate and TNF inhibition in rheumatoid arthritis

**DOI:** 10.1186/ar3756

**Published:** 2012-03-05

**Authors:** Altan Ercan, Jing Cui, Melissa M Hazen, Franak Batliwalla, Louise Royle, Pauline M Rudd, Jonathan S Coblyn, Nancy Shadick, Michael E Weinblatt, Peter Gregersen, David M Lee, Peter A Nigrovic

**Affiliations:** 1 Division of Rheumatology, Immunology and Allergy, Brigham and Women's Hospital, One Jimmy Fund Way, Smith 516c, Boston MA, 02115 USA; 2 Division of Immunology, Children's Hospital Boston, 300 Longwood Avenue, Boston, MA 02115, USA; 3 Robert S Boas Center for Genomics and Human Genetics, Feinstein Institute Medical Research, 350 Community Drive, Manhasset, NY 11030, USA; 4 Ludger Ltd, E1 Culham Science Centre, Abingdon, Oxfordshire, OX14 3EB, UK; 5 Dublin-Oxford Glycobiology Laboratory, National Institute for Bioprocessing Research and Training (NIBRT), Conway Institute, University College Dublin, Belfield, Dublin 4, Ireland; 6 Novartis Institute for Biomedical Research (NIBR) Autoimmunity, Transplantation and Inflammation (ATI) CHBS, WSJ-386.11.05, Novartis Pharma AG, Novartis Campus, CH-4056 Basel, Switzerland

## Abstract

**Introduction:**

Rheumatoid arthritis (RA) is associated with hypogalactosylation of immunoglobulin G (IgG). We examined whether a proxy measure for galactosylation of IgG N-glycans could predict response to therapy or was differentially affected by methotrexate (MTX) or TNF blockade.

**Methods:**

Using a previously defined normal phase high-performance liquid chromatography approach, we ascertained the galactosylation status of whole serum N-glycans in two well-defined RA clinical cohorts: the Autoimmune Biomarkers Collaborative Network (*n *= 98) and Nested I (*n *= 64). The ratio of agalactosylated to monogalactosylated N-glycans in serum (sG0/G1) was determined before and during therapy with MTX or TNF inhibition and correlated with anticitrullinated peptide antibody (ACPA) status and clinical response as assessed by 28-joint Disease Activity Score utilizing C-reactive peptide and European League Against Rheumatism response criteria.

**Results:**

RA patients from both cohorts exhibited elevation of sG0/G1 at baseline. Improvement in clinical scores correlated with a reduction in sG0/G1 (Spearman's ρ = 0.31 to 0.37; *P *< 0.05 for each cohort). However, pretreatment sG0/G1 was not predictive of clinical response. Changes in sG0/G1 were similar in the MTX and TNF inhibitor groups. Corrected for disease activity, ACPA positivity correlated with higher sG0/G1.

**Conclusions:**

Baseline serum N-glycan hypogalactosylation, an index previously correlated with hypogalactosylation of IgG N-glycans, did not distinguish patients with rheumatoid arthritis who were likely to experience a favorable clinical response to MTX or TNF blockade. Clinical improvement was associated with partial glycan normalization. ACPA-positive patients demonstrated enhanced N-glycan aberrancy compared with ACPA-negative patients.

## Introduction

Human immunoglobulin G (IgG) is a glycoprotein with a biantennary (that is, two-armed) oligosaccharide attached to a canonical asparagine (N) in each heavy chain. These N-glycans are unusual because they do not decorate the protein surface. Instead, they are largely enclosed within the Fc region, where they help to maintain its spatial conformation. Variations in glycan structure "fine-tune" the effector activity of the antibody, modulating its capacity to fix complement and engage Fc receptors [1,2]. Certain Fc glycan variants enriched for terminal sialic acid render IgG overtly anti-inflammatory, accounting in part for the action of high-dose intravenous Ig [3,4].

Interestingly, rheumatoid arthritis (RA) is characterized by alterations in IgG glycosylation [5-8]. Patients with RA exhibit an elevated proportion of IgG in which neither of the two glycan arms bears a terminal galactose (thus termed "G0"). This conformation enables binding of mannose-binding lectin, resulting in an enhanced propensity to fix complement, and animal studies suggest that G0 IgG may be especially arthritogenic [9-11]. Recently, we and others have confirmed this hypogalactosyl phenotype in large cohorts, demonstrating additionally that change in IgG glycosylation predates the diagnosis of RA by an average of more than 3 years, is enriched in antibodies directed against citrullinated peptides (ACPAs) and correlates with disease activity [12-16]. Thus multiple lines of evidence point to a role for IgG glycans in the pathogenesis of RA.

Although RA patients as a group exhibit altered IgG glycans, there remains substantial heterogeneity within this population [15]. We wished to understand whether pretreatment glycan status could predict response to therapy and whether disease-modifying antirheumatic drugs (DMARDs) might affect glycans differently, potentially hinting at an unexplored mode of action. Furthermore, we wished to determine whether ACPA positivity correlated with IgG glycoform aberrancy. We therefore performed an analysis of whole-serum N-glycan galactosylation, previously noted to correlate highly with galactosylation of IgG N-glycans [15,17], on serial samples collected prospectively from patients with RA before and after treatment with MTX and anti-TNF agents.

## Materials and methods

### Patients

Patient samples were obtained from two cohorts, both of which have previously been described in detail [18,19]. The Autoimmune Biomarkers Collaborative Network (ABCoN) enrolled RA patients with at least six swollen joints who received 10 mg or less of prednisone at the initiation of TNF inhibitor therapy. Nested I employed identical entry criteria at the initiation of therapy with either MTX or a TNF inhibitor. The patients were allowed to add an additional agent after 6 weeks. Approximately 60% of TNF starters in each cohort received concomitant MTX at a stable dose. In both cohorts, serum samples were collected at baseline and 3 months after initiation of treatment. In Nested I, serum was also collected after 2 weeks. Disease activity was assessed using the 28-joint Disease Activity Score using CRP (DAS28-CRP) at baseline and at 3 months. ACPA status was assessed using the QUANTA Lite CCP IgG ELISA kit, version 2 (a second-generation ACPA assay, INOVA Diagnostics, Inc, San Diego, CA, USA). ABCoN and Nested I patients provided their written informed consent for sample acquisition. Healthy adult control samples were obtained from deidentified blood donors as described previously [15]. All samples were acquired with the approval of the respective institutional review boards.

### Glycan characterization

Glycans were analyzed as described in detail previously [15,17]. Briefly, N-glycans were liberated enzymatically from 5 μl of whole serum, labeled and analyzed by normal-phase high-performance liquid chromatography (NP-HPLC), which provides precise relative quantitation of molecular species separated by size and charge. The area under the glycan elution peaks was calculated, and G0 was normalized to the monogalactosylated (G1) fraction, which remains relatively constant across the population [20,21]. Because the majority of neutral biantennary glycans in serum are derived from IgG, the ratio of agalactosylated to monogalactosylated N-glycans in serum (sG0/G1) has been employed as a proxy for IgG G0/G1 (*R*^2 ^= 0.83) [15,17].

### Statistical analysis

Population means were compared using Student's *t*-test (two-tailed), paired or unpaired as appropriate. Correlations between change in sG0/G1 and DAS28-CRP were assessed by Spearman's ρ coefficients. Statistical analyses were performed using GraphPad Prism version 4.0 software (GraphPad Software, Inc, La Jolla, CA, USA) or SAS version 9.2 software (SAS Institute, Cary, NC, USA).

## Results

### sG0/G1 glycan ratio is altered at baseline and improves with treatment

**Figure 1:**
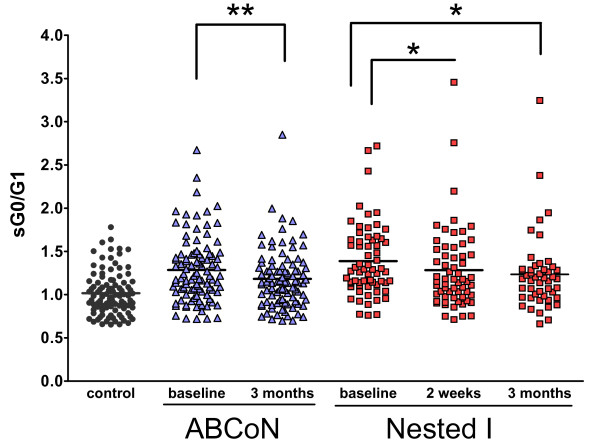
**Ratio of agalactosylated to monogalactosylated N-glycans in serum immunoglobulin (sIgG0/G1) is elevated at baseline and improves with treatment**. Data show sG0/G1 from baseline and follow-up visits in both cohorts. All rheumatoid arthritis time points *P *< 0.001 vs healthy controls. *P*-values were derived from paired *t*-tests (*P < 0.05; **P = 0.01). Paired data available for Autoimmune Biomarkers Collaborative Network were *n *= 75 vs 3 months; Nested I, *n *= 61 vs 2 weeks and *n *= 48 vs 3 months.

**Figure 2:**
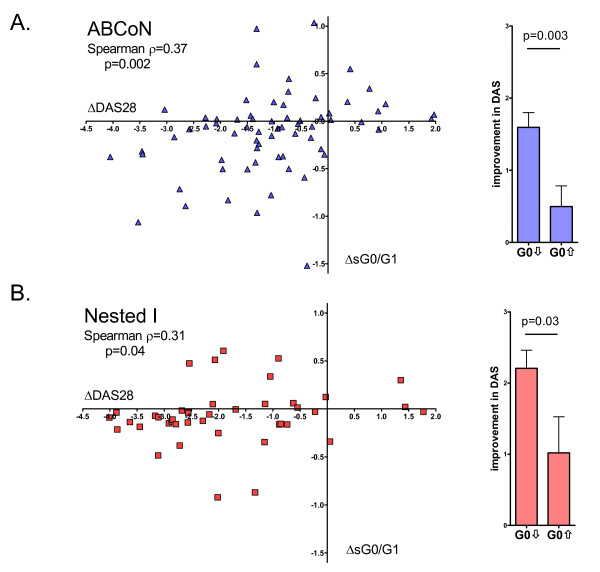
**Correlation between changes in ratio of agalactosylated to monogalactosylated N-glycans in serum (sG0/G1) and 28-joint Disease Activity Score utilizing C-reactive peptide**. Differences reported are between baseline and 3-month visits. Each point represents an individual patient. Plots at right depict improvement (decrease) in Disease Activity Score (DAS) comparing patients in whom the sG0/G1 either increased or decreased appreciably (> 0.1 U) over the interval studied. **(A) **Autoimmune Biomarkers Collaborative Network (ABCoN). **(B) **Nested I.

Glycan profiling was performed on the sera of 98 ABCoN patients, 64 Nested I patients and 102 matched controls (Table 1). In the ABCoN cohort, patient therapy was initiated with a TNF inhibitor (42 etanercept, 32 infliximab and 24 adalimumab). In the Nested I cohort, 34 patients received anti-TNF therapy (26 etanercept, 1 infliximab and 7 adalimumab) and 30 patients received MTX. Clinical severity assessed 3 months after recruitment declined in both cohorts, with mean (± SD) DAS28-CRP scores declining from 5.16 ± 0.95 to 3.72 ± 1.33 in the ABCoN group (*P *< 0.0001) and from 5.62 ± 0.91 to 3.82 ± 1.55 in the Nested I group (*P *< 0.0001). As expected, sG0/G1 ratios were high at baseline and declined after 3 months of therapy, though they remained abnormal (Figure 1). Reduction in sG0/G1 ratios paralleled clinical improvement, as demonstrated by the correlation between the changes in sG0/G1 and DAS28-CRP in both cohorts (Figure 2).

**Table 1:** Patients analyzed^a^

Patient characteristics	ABCoN (*N *= 98)	Nested I (*N *= 64)	Control (*N *= 102)
Mean (SD) age, years	54.8 (12.8)	52.6 (13.3)	55.9 (13.0)
Sex (female), *n *(%)	75 (78)^b^	53 (83)	77.4
Mean (SD) disease duration, years	10.3 (9.0)	9.2 (11.6)^b^	NA
Mean (SD) disease activity, DAS28 score	5.16 (0.95)	5.62 (0.91)	NA
RF-positive, *n *(%)	81 (87.1)	45 (70.3)^b^	NA
ACPA-positive, *n *(%)	75 (80.5)	37 (74.0)^b^	NA
Mean (SD) sG0/G1	1.37 (0.42)	1.38 (0.41)	1.02 (0.25)

^a ^ABCoN, Autoimmune Biomarkers Collaborative Network; ACPA, Anti-Citrullinate Peptide Antibody; DAS28, 28-joint Disease Activity Score; RF, rheumatoid factor; sG0/G1, ratio of agalactosylated to monogalactosylated N-glycans in serum. ^b^Fractions represent proportions within available data. ABCoN gender data available in 96 patients; Nested I, disease duration available in 62 patients, RF in 63 and ACPA in 50 patients.

### Serum N-glycan hypogalactosylation is not a predictor of response to treatment

**Figure 3:**
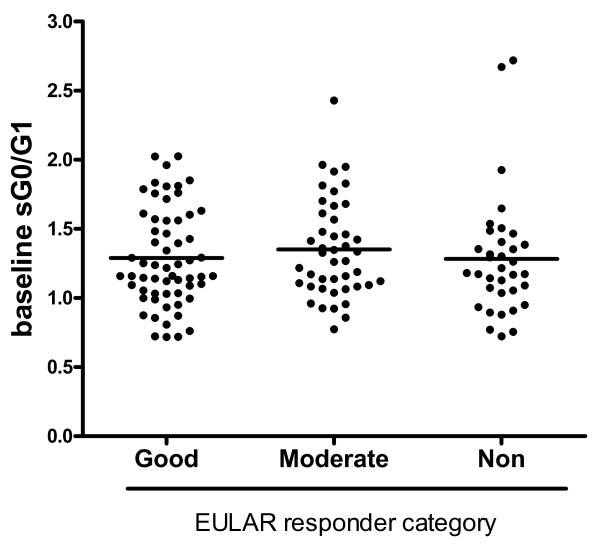
**Ratio of agalactosylated to monogalactosylated N-glycans in serum (sG0/G1) does not predict therapeutic response**. Baseline sG0/G1 stratified according to European League Against Rheumatism (EULAR) response category at 3 months polled from both Autoimmune Biomarkers Collaborative Network and Nested I cohorts. Mean ± SD for good responders 1.29 ± 0.35, moderate 1.35 ± 0.35, nonresponders 1.28 ± 0.44. *P *= ns for all comparisons.

Because reliable predictors of response to therapy remain elusive, we examined whether baseline sG0/G1 status correlated with clinical improvement upon treatment. For this analysis, we stratified patients after 3 months of therapy into good responders (GRs), moderate responders (MRs) and nonresponders (NRs) according to European League Against Rheumatism response criteria [22]. No differences in baseline sG0/G1 ratio were observed (Figure 3). Using the Nested I data at 2 weeks, we further considered whether early changes in sG0/G1 might predict response to therapy at 3 months, sparing patients from unnecessary exposure to expensive and potentially toxic therapy. However, similar nonsignificant declines in sG0/G1 were observed in all groups. The mean changes (SD) were -0.11 (0.27) for GRs, -0.23 (0.50) for MRs and -0.11 (0.15) for NRs. Acknowledging the caveat that the half-life of IgG is approximately 23 days [23], such that an analysis at 2 weeks might be too soon to note substantial changes, we found no suggestion that change in sG0/G1 could be used as an early marker of therapeutic efficacy.

### Effect of therapy with TNF inhibition or methotrexate on sG0/G1

**Figure 4:**
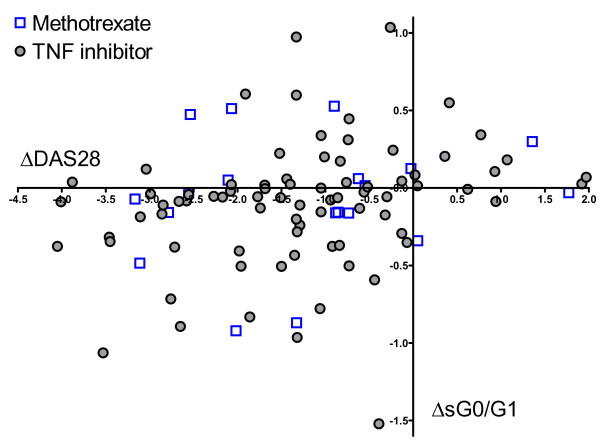
**Change in ratio of agalactosylated to monogalactosylated N-glycans in serum (sG0/G1) with therapy does not distinguish methotrexate (MTX) and TNF inhibition**. Relationship between therapy and the correlation between clinical improvement and changes in sG0/G1 for patients in whom MTX or TNF inhibition was the only new therapeutic agent in the interval between t = 0 and t = 3 months. TNF inhibitor (*n *= 79) (Autoimmune Biomarkers Collaborative Network and Nested I), Spearman's ρ = 0.34; MTX (*n *= 19) (Nested I only), Spearman's ρ = 0.16 (*P *= 0.56 vs TNF inhibitor group).

If hypogalactosylated IgG is pathogenic, then IgG glycosylation might represent an unrecognized target of DMARD therapy. Two of the most commonly used therapies for RA are MTX and TNF inhibition. These classes of agents are therapeutically complementary and attain their efficacy via complex and incompletely understood pathways. Human B cells express several different receptors for adenosine, an anti-inflammatory mediator implicated in the mechanism of action of MTX [24,25]. B cells also express receptors for TNF, and treatment of RA with TNF inhibitors has been shown to result in disruption of germinal centers [26,27]. No data are available on the impact of either pathway on IgG glycosylation. We considered the possibility that either MTX or TNF inhibition might exert an anti-inflammatory effect in part via modulation of IgG glycans, rendering autoantibodies less pathogenic. We therefore examined total serum N-glycan patterns in patients receiving these therapies to ascertain whether the correlation between clinical improvement (change in DAS-CRP) and improvement in sG0/G1 differed between agents. However, no such difference was noted (Figure 4).

### Anticitrullinated peptide antibody status as a predictor of sG0/G1

Several groups have shown that ACPA IgG may exhibit unusually low levels of N-galactosylation [15,16]. To date, however, no association between ACPA status and level of total IgG galactosylation has been defined. Recognizing that sG0/G1 varies with disease activity, we assessed the relationship between ACPA status (positive or negative) and aberrant sG0/G1 corrected for DAS28-CRP. In pooled baseline data from ABCoN and Nested I, we were able to establish a significant association between ACPA positivity and higher sG0/G1 values (*P *= 0.003). Pooled analysis with our recently published cohort of 292 RA patients [15] strengthened this association (*P *< 0.0006).

## Discussion

Abnormal glycosylation of IgG is a well-established phenotype in RA, yet its clinical relevance is uncertain. In this study, we employed a proxy measurement for IgG galactosylation to address two key questions within the glycobiology of RA: (1) whether IgG glycan aberrancy predicts response to treatment and (2) whether DMARDs exert differential impacts upon IgG glycosylation, thereby potentially contributing to their clinical effectiveness. Using a well-characterized high-throughput NP-HPLC technique, we examined total serum N-glycans in two cohorts of RA patients for whom serial blood samples and prospectively collected clinical data were available. Because most biantennary serum N-glycans originate from IgG, our data support prior work indicating that IgG is hypogalactosylated in patients with RA and that a direct correlation between clinical and glycan improvement can be observed at the level of the individual patient. However, we found no evidence that (1) baseline sG0/G1 or change in sG0/G1 two weeks after initiation of treatment could predict therapeutic response to TNF inhibition or MTX or (2) these agents differentially influenced N-glycan galactosylation. The latter result suggests either that these agents exert similar effects on IgG glycans or, perhaps more likely, that altered IgG galactosylation arises via another route in patients experiencing clinical improvement.

By contrast, herein we show for the first time a clear correlation between ACPA positivity and abnormal galactosylation. This correlation had not previously been observed, likely because of confounding by differential disease activity. Our result therefore corroborates the observation of several groups that IgG ACPAs may exhibit glycosylation that is more aberrant than that of the IgG pool as a whole [15,16].

These results highlight gaps in the understanding of IgG glycobiology in RA. The glycosylation pattern of IgG reflects multiple factors, perhaps most directly the cellular complement of specific enzymes that catalyze each step in glycan assembly [28-30]. Patients with RA show reduced peripheral blood B-cell galactosyltransferase activity, though the biological basis for this difference and its relevance for IgG production by plasma cells has not been defined [31-33]. How disease activity is associated with altered glycosylation, why this relationship differs from patient to patient (see, for example, Figure 2) and how antibodies of different specificities can display distinct glycan profiles remain to be defined. In this context, recent work illustrating particular glycan aberrancy of ACPAs from synovial fluid is of interest because it suggests that hypogalactosylated IgG may be generated directly within the rheumatoid synovium, accounting perhaps for the decline of sG0/G1 as this microenvironment normalizes with treatment [16].

Our study has several limitations. As noted in the Materials and methods section, the sG0/G1 index provides an imperfect estimate of IgG G0/G1. We cannot exclude the possibility that some of the changes observed reflect serum glycoproteins beyond IgG, though we are reassured in this respect by the work of other authors who have observed similar changes in IgG purified from RA patients [12-14]. In particular, because IgG is the major serum source of G0 and G1 glycans, we doubt that we would have missed a clinically important predictive role of IgG galactosylation while using our assessment technique, though formal confirmation of this conclusion would require analysis of purified IgG.

Furthermore, we recognize that our method captures only part of the complexity of IgG Fc glycans. In addition to galactosylation, IgG glycans can vary in core fucosylation, the presence of an additional N-acetylglucosamine bisecting the two glycan arms and the extent of terminal sialylation. Each of these variants can have functional implications, modulating the ability to fix complement, engage Fc receptors and serve as a ligand for the myeloid lectin receptor dendritic cell-specific intercellular adhesion molecule 3-grabbing nonintegrin, or DC-SIGN [1,2]. Therefore, our data do not exclude the possibility that further examination of individual IgG glycoforms, perhaps together with other clinical or genetic markers, might still uncover associations of diagnostic or therapeutic importance in RA. Because patients receiving MTX alone were enrolled only in the smaller cohort (Nested I), relatively few were available for analysis, thus limiting the power of our data to exclude definitively a difference between MTX and TNF inhibitors with respect to the correlation between changes in G0/G1 and in DAS (Figure 4).

## Conclusions

These results represent the first exploration of the potential utility of IgG glycosylation as a predictor of therapeutic response in RA. Our results confirm that hypogalactosylation of IgG (assessed in our present study via the proxy measure sG0/G1) is prominent in patients with RA and improves with therapy, but they neither support the use of the sG0/G1 index as a guide to choosing between MTX and TNF inhibitors nor suggest a differential effect of these agents on IgG galactosylation. However, we have identified for the first time an association between ACPA positivity and aberrant IgG glycosylation, further extending the understanding the relationship between antibody glycans and RA.

## Abbreviations

ABCoN: Autoimmune Biomarkers Collaborative Network; ACPA: anticitrullinated protein antibody; DAS28-CRP: 28-joint Disease Activity Score utilizing C-reactive peptide; DMARD: disease-modifying antirheumatic drug; HPLC: high-performance liquid chromatography; MTX: methotrexate; RA: rheumatoid arthritis; sG0/G1: ratio of agalactosylated to monogalactosylated N-linked biantennary glycans in serum; TNF: tumor necrosis factor.

## Competing interests

The authors declare that they have no competing interests.

## Authors' contributions

AE participated in study design and coordination, carried out the glycan analysis and participated in data interpretation and manuscript preparation. JC participated in the statistical analysis. MMH participated in sample acquisition and glycan analysis. LR and PMR participated in glycan analysis and data interpretation. FB, JSC, NS, MEW, and PG participated in sample acquisition. DML participated in study design and coordination. PAN participated in study design and coordination, directed data analysis, and drafted the manuscript. All authors read and approved the final manuscript.
